# Using of Mystery Clients’ Experiences and Feedback to Improve the Quality of Family Planning Services in Northern Ghana: Evidence From an Uncontrolled Quasi-experimental Study

**DOI:** 10.7759/cureus.90209

**Published:** 2025-08-16

**Authors:** Naa-Korkor Allotey, Evans K Appiah, Kwasi Torpey

**Affiliations:** 1 Ethics and Research Management, Research and Development Division, Ghana Health Service, Accra, GHA; 2 Disease Control, Tolon District Health Directorate, Tolon, GHA; 3 Population, Family and Reproductive Health, School of Public Health, University of Ghana, Accra, GHA

**Keywords:** family planning services, health facilities, health workers, mystery clients, northern ghana, quality

## Abstract

Introduction

Although there is a global increase in modern contraceptive uptake, some countries, including Ghana, have stalled, particularly in the northern part of the country. One contributory factor is the quality of family planning services provided at health facilities. Conventional methods used to assess the quality of care in health facilities may not be effective in identifying quality issues in areas like reproductive health. Nevertheless, mystery client (MC) surveys and feedback have been used globally to identify sensitive issues that conventional methods may have missed. However, there is limited evidence of their use in family planning services in Ghana. This study, therefore, aimed to use the MC approach with feedback to improve the quality of family planning service delivery based on specific actionable points in Northern Ghana.

Methods

Three rounds of mystery client surveys were completed in a quasi-experimental design with no control group in eight health facilities in 2019. Different sets of mystery clients were used, and feedback was given to health workers after each survey. Quality of care was graded using 13 actionable points. These actionable points include "Warm reception", "Confidentiality and Privacy", "Service available 24/7", "Identifying wandering clients", "Availability of preferred commodity", among others. The total score for each health facility was calculated, and the mean score for the eight facilities was determined. T-test and repeated-measures ANOVA were used to determine whether service improvement was significant. Minutes of feedback sessions were documented, and content analysis was done to identify relevant themes and codes.

Results

Grading for the three rounds of the mystery client surveys showed consistent service improvement for successive rounds. The mean scores of the quality of family planning services increased from 20.0 (±6.9) to 30.4 (±6.2), and the p-value is 0.0032. Some identified themes from the content analysis of the feedback sessions included "risk of identification of MCs by health workers", "reactions of health workers to their performance", and "district health authorities' decision to adopt the MC approach into their monitoring strategies".

Conclusion

Using successive mystery client surveys with the associated feedback based on specific actionable points improved the quality of family planning services. Decision makers should incorporate mystery client surveys with feedback in their monitoring strategies to identify sensitive issues that would be missed using conventional methods.

## Introduction

The importance of family planning (FP) in improving the health and social outcomes of women and their children has been well documented [1,2]. However, the uptake of modern contraception continues to stall in parts of Africa despite a general global increase in family planning uptake [3]. Challenges in uptake are due to several factors from both the supply and demand sides. Factors such as attitude, awareness, and knowledge create demand for modern contraception. Supply-side factors that influence modern contraception include accessibility to health facilities, availability of transportation, and resources such as FP commodities [4].

In Ghana, there has been a stall in the contraceptive uptake, particularly in Northern Ghana. For example, trends in the modern contraceptive prevalence rate (mCPR) were 22.1% in 2003, 21.1% in 2008, 20.4% in 2014, and 22% in 2017 [5]. In 2018, the Northern Region recorded 17% mCPR, the lowest in Ghana [6]. Various efforts have been made to improve the demand and supply sides of contraceptive uptake [4,7-8]. Some supply-side interventions to improve contraceptive uptake include increasing contraceptive availability in communities [9] and capacity-building of health workers [10].

The quality of services provided by health professionals is another key factor in improving service uptake [11]. When the quality of services is perceived to be poor, it negatively influences the utilization of health services [12]. For instance, Obssa et al used a quality improvement approach to identify gaps in immediate post-partum family planning (IPPFP) services, after which they proposed solutions such as "improvement in quality of counseling", "timely request and refill of contraceptive commodities", among others. Implementation of the proposed solutions helped to improve IPPFP in four facilities from 21% to 69% [13]. Additionally, Mwaniki et al investigated the potential of applying improvement strategies to enhance the utilization of antenatal care (ANC) services, health facility deliveries, among others. They found that ANC registrants within the first trimester increased from 8% to 24%, p=0.002, and health facility delivery increased from 33% to 52%, p=0.012 [14]. 

Generally, an assessment of the quality of services rendered to clients is conducted using data collection methods such as exit interviews or supportive supervision with feedback to the healthcare workers [15]. However, several authors acknowledge that some pertinent quality issues are not identified using traditional methods such as exit interviews and supportive supervision. For instance, a systematic review conducted by Aujla et al documented that healthcare workers’ responses in exit interviews may be influenced by several biases, such as social desirability bias, recall bias, and courtesy bias. Potentially, these biases lead to an overestimation of the perceived quality of care [16].

The use of mystery clients (MCs), however, has been useful in identifying issues that would otherwise have been missed by other data collection methods [17,18]. Mystery client data collection methods have been used in several reproductive health studies. For example, Ganle et al [17] resorted to using mystery clients in their study to obtain accurate information on the illegal availability of abortifacients in pharmacies in Ghana. They found that responses from questionnaires showed that 50.3% of pharmacies reported using abortifacients, while 74% of pharmacies were found to have abortifacients using the mystery client approach. Findings from Ganle et al show that about 24% of pharmacies failed to report the availability of arbotificients because of the illegality of selling them in pharmacies [17]. In this regard, Ganle et al found that institutions do not report certain sensitive issues linked to illegalities that take place in their workplaces. 

Mystery client data collection methods are employed for several other reasons. Firstly, MCs can interact with employees or health workers to get information that would not have been obtained through other methods. Secondly, the method can be used without interrupting the course of service provision. Moreover, mystery client surveys also identify problems that would have otherwise been missed [19].

There are, however, drawbacks to the use of MC methods. One of the limitations of using mystery clients is that repeated clients run the risk of being identified. MCs must also have a strong ability to recall the services received. Furthermore, detailed training protocols are needed to ensure that MCs mimic real clients. Protocols should, however, prevent MCs from being examined unnecessarily [18].

Based on the argument above, it is recognized that other methods are used to evaluate the quality of family planning services. However, these methods lack the ability to identify sensitive issues regarding service delivery processes, particularly when the healthcare worker is working in their normal environment. Despite some disadvantages, the MC approach helps to fill the gaps in the traditional monitoring methods. Therefore, this study aimed to use the MC approach with feedback to improve the quality of family planning service delivery based on specific actionable points in Northern Ghana. 

## Materials and methods

Study design

The study employed a quasi-experimental design with no control group, incorporating mystery client surveys with feedback provided to health workers from the participating health facilities.

Study setting

The study was conducted in Tolon District, one of the districts in the Northern Region of Ghana. Tolon District is about 440 km from the national capital, Accra. The major economic activity in the district is agriculture. The district has six sub-districts: two urban and four rural sub-districts. One urban and one rural sub-district were randomly selected (based on the assessment of district health authorities). According to the 2021 National Population and Housing Census (PHC), the population of Tolon District was 118,101 [20]. Almost all citizens are Dagombas and are Muslims. There are three health centres, 10 Community-Based Planning Health Services (CHPS) facilities, and one community clinic. The health facilities involved in the study were two health centres, a clinic, and five CHPS facilities (similar to health posts, and the lowest type of health facility in the Ghanaian health system). These health facilities were all the facilities in the sub-districts involved in the study. Additionally, two health facilities (HC2 and CHPS5) in adjacent sub-districts of the selected sub-districts were included in the study based on their proximity to the participants of the larger study. These two facilities were not included in the initial training for the health workers.

Ghana has a four-tier health system made up of teaching hospitals at the apex of the system, regional hospitals as tier three, district hospitals as tier two, and health centres, clinics, and CHPS as tier one. Under tier one, however, there are various levels, with health centres having a higher level of staff. These health centres are staffed by physician assistants, midwives, registered nurses, and other lower-calibre staff compared to CHPS facilities, which are mainly staffed by community health nurses, who generally have a lower qualification. A clinic is considered an intermediary between the health centre and CHPS. No clinic met the criterion of accessibility. To ensure anonymity and privacy, the names of the health facilities that were studied were given codes (e.g., HC1 for health centre and CHPS1, etc.)

Study participants and sample size

The study population consisted of all health facilities within the two sub-districts selected for the larger study. In addition, two facilities were included, based on accessibility to the study participants of a larger study. In all, eight facilities were included in the study. Hence, a census of all facilities that were accessible to the participants of the larger study was used. The rationale for selecting these facilities was to ensure that participants in the larger study would receive quality services based on the specific actionable points. 

Intervention description and data collection

Summary of Interventions for a Larger Study: The MC surveys were part of interventions of a larger study aimed at persuading couples to use modern contraceptives in Northern Ghana. The following sets of interventions used in the larger study were: (1) Intrapersonal and interpersonal level: Couples of the larger study participants had meetings and were counselled; (2) Community level: There was a screening of a two-part film on the benefits of modern contraceptives in intervention communities; (3) Organisational level: This entailed the orientation of health workers, conducting the MC surveys, and giving feedback to health workers.

Orientation of Health Workers: We started the organisational intervention in February 2019 with the orientation of health workers in the intervention health facilities. We opted to orient health workers rather than train them because all the health workers were practicing family planning service providers and had been previously trained.

Staff were divided into two groups to ensure that family planning services were not interrupted in the health facilities. Thus, the second group had their training immediately after the first training had ended. Each group went through a two-day orientation. The training included discussions on the tenets of family planning and the quality of care given to clients. The training also had role-play sessions. The training was interactive, emphasizing the need to improve the practices of the health workers. At the end of the training, 13 actionable points were collectively agreed upon between the health workers, the facilitators, and the investigative team. It was also agreed that these actionable points would be adopted into routine practice to improve the quality of family planning services. The following were the actionable points: warm reception, offering the client a seat, confidentiality, de-identifying FP rooms, service available 24/7, identifying wandering clients, removing the barrier of cost of urine pregnancy test (UPT), any service provider can offer services, ruling out pregnancy, documentation, in-depth commodity explanation, availability of preferred FP method, and information that FP prevents pregnancy but not STIs. The points were printed on A3 sheets, laminated, and distributed to the eight participating health facilities.

The health workers also consented at the training that they would be visited by MCs to monitor the quality of service. However, the health workers were not informed about the specific day that the MC surveys would take place. The district health authorities also agreed with this arrangement. 

Recruitment and training of mystery clients

Recruitment: In Round 1, a member of the research assistants in the larger study was tasked to invite her colleagues to be recruited as MCs. Ten student nurses were initially recruited and trained, but three of the highest performing recruits were selected as MCs. We observed from the first feedback session that the student nurses could be identified by the health workers because they did not resemble their usual clients. Therefore, we resorted to using women from the participating district. In Round 2, community health volunteers from outside the participating communities of the larger study were tasked to identify five women from their communities. Permission was then sought from their husbands, after which they were trained. In Round 3, it was difficult to get women from communities outside the communities for the larger study as a result of stigma. In this case, we resorted to using women from the larger study. 

Training: The three sets of mystery clients were trained with one protocol, with the same scenario. The scenario was scripted; however, the MCs did not visit the facilities with the script. The mystery clients were trained such that they could act out the scenario without needing the script. The scenario took cognisance of all 13 actionable points. There were role-plays to ensure that the MCs understood the scenario and knew what to say and how to act. The scenario was as follows: A female client had a child and did not want to become pregnant anytime soon. The client must be new (first time to use FP). The MC was expected to wander at the peripheries of the health facility, reluctant to come into the facility and hoping to catch the eye of a health worker. If she waited too long (10 to 15 minutes) and was not noticed by any health worker, she would then approach a staff member. The client was to establish that she was not from the community. After approaching the health worker, MC stood to see if the health worker would offer a seat and provide a warm reception. The health worker was further observed to see whether he would send the MC to a de-identified room (a room not labelled with “Family Planning”). The health worker attending to the client was also observed to see if they would turn the client away if she requested a specific health worker or if the care provider would persuade her to receive the services to avoid a missed opportunity. It was further observed to see if clients were turned away by the health worker because they could not pay for the cost of a pregnancy test. The health worker was, however, expected to rule out pregnancy and also to inform the client that only condoms could prevent sexually transmitted infections (STIs). Furthermore, the health worker was expected to give in-depth information on the different family planning methods. The healthcare provider was expected to offer the client her preferred family planning method (MCs were trained to opt for combined oral contraceptives (COCs) because COCs are a non-invasive method). Finally, the provider was expected to inform her that services were available 24 hours a day, seven days a week. 

Mystery Client Surveys: Three rounds of mystery client surveys were conducted between April and August 2019. Round 1 survey was done in April 2019, two months after the orientation of the service providers. Round 2 took place on 2nd July 2019, and Round 3 was conducted on 29th August 2019. It must be acknowledged that the period between Rounds 2 and 3 was shorter than the originally stipulated 3 months, largely because the intervention period for the larger study was ending. The three different MC surveys were used as a monitoring tool to assess the quality of family planning services given to clients. 

In Round 1, three female student nurses who had completed pre-service training but had not started work acted as mystery clients. They were given a day’s training and were made to role-play the scenario. Each was given a mobile phone with a recorder. The mystery clients were asked to report and narrate proceedings after they had received the services from the health workers. The conversation with the health workers was not recorded during service provision for ethical reasons, but MC gave an overview and narrative of what transpired when she was receiving services immediately after she stepped out of the health facility premises to reduce recall bias. The MCs did the recording before they moved to the next facility.

Round 2 used women from communities outside the intervention areas who served as MCs, while Round 3 involved female study participants (from the larger study) who served as mystery clients. In the subsequent rounds, however, the MCs did not do any audio recordings of their observations after services had been provided for them. District-level officers intentionally coincided with the MCs to observe the proceedings and graded the facility while the MC was receiving an FP service from the health worker. Different calibres of MCs were used for the various rounds in a bid to avoid being identified by health workers. Each MC was assigned two or three facilities. A visit to a facility by the MC took an average of 2 hours.

Tool and data collection

The list of actionable points was converted into a checklist, which was put in a tabular format (see Appendices). The first column of the checklist contained the serial number of each actionable point. The second column displayed a list of actionable points, while the third column was used for grading the quality of family planning services. A copy of the checklist was filled out for each facility. This checklist uses a routine grading system used by the Ghana Health Service. The grading was as follows: 0, 1, 2, and 3. That is, 0 or 1 was the lowest score, and 3 was the highest score. Where 0 indicated “No action performed”, 1 represented “poorly performed action”, 2 represented "fairly performed action”, and 3 represented “good action performed”. In Round 1, the student nurses’ audio recordings were listened to and graded by district health authorities. In Rounds 2 and 3, non-core district health authorities (an experienced midwife and a reproductive and child health officer), under the guise of ‘passing by and deciding to visit the health facilities', observed the interaction between the MC and the health worker and graded the facilities while they pretended not to know the MCs. We selected non-core district health authorities because the MCs used in Rounds 2 and 3 were illiterate. The investigative team, therefore, determined that using assessors to document proceedings would ensure greater accuracy. 

Feedback

After each round of the survey, feedback was given to the health workers at staff meetings by the investigative team and the district health management team. Presentations were made on the performance of the facilities by generating a league table (a league table is defined as the ranking of the facilities based on the scoring of each actionable point) for each round of the MC survey. These presentations enabled health workers in each facility to recognise their performance on each actionable point. The possible reasons for each facility's performance were discussed, and solutions were proposed and agreed on between the health workers and district authorities. Minutes of the three staff meetings were taken. The district health authorities later reviewed the minutes to identify issues of concern that were consistently raised. These issues were incorporated into the district's routine monitoring checklist.

Furthermore, as a means of coaching and follow-up, the district health authorities visited the facilities and discussed the performance of the previous MC survey and how to improve FP services. 

Data management and analysis

The district health authority and investigative team collaboratively retrieved the collected data, conducted quality assurance checks, and ensured that grading was carried out as objectively as possible. The data collected on the checklist was extracted and collated on the league table for all facilities. The different rounds were compared for each health facility and each actionable point. Using the Bruce categorisation, the actionable points were grouped based on Bruce’s framework, which consists of six tenets: choice of methods, the information given to clients, technical competence, interpersonal relations, follow-up and continuity mechanisms, and an appropriate constellation of services. We applied five of the six tenets because Bruce’s framework assesses the quality of health services specific to family planning from the client’s perspective. Additionally, the actionable points aligned more effectively with Bruce’s categorisation compared to other quality assessment frameworks [21]. The actionable points were grouped as follows: warm reception and offering the client a seat (interpersonal relations), confidentiality, de-identifying FP rooms, service available 24/7, identifying wandering clients, removing the barrier of cost of urine pregnancy test (mechanisms to encourage contraceptive continuation), any service provider can offer services, ruling out pregnancy, documentation (technical competence), in-depth commodity explanation, availability of preferred FP method (choice of methods), and FP prevents pregnancy but not STIs (Provider: questions asked and information given). Percentages were calculated for the various categories for each round by the sum of all health facility scores for the actionable points in the respective categories divided by the maximum score for each category for all eight health facilities, and multiplying the results by hundred. Each actionable point had a maximum possible score of 3. With a total of 13 actionable points, the highest attainable score was 39.

To assess improvements in the quality of care, the mean scores and standard deviation for the facilities were calculated for each round of the mystery client (MC) survey. This was done by summing the scores of all actionable points across all facilities for each survey round. The mean score for each MC round was then obtained by dividing the total score by 8, representing the number of health facilities involved. A t-test was used to compare the means between consecutive rounds of the MC survey (that is, the mean score for Round 1 was compared with Round 2, Round 2 compared with Round 3, and Round 1 compared with Round 3). An analysis of variance (ANOVA) was used to compare the three rounds. The level of significance was assessed by p-values. These tests were based on recommendations by Khan and Adil [22]. Additionally, normality, independence, and equality of variance of the dataset were tested using the Shapiro-Wilk test, scatter plots (shown in Figure 3), and Mauchly's test, respectively.

Qualitative analysis of feedback sessions

Content analysis was used to analyze the minutes of the feedback sessions. Themes and codes were identified. 

## Results

Table 1 shows the background characteristics of health workers in participating health facilities. It was observed that the mean age of health workers at the health centres was 39.2 years, while the mean age of health staff at the CHPS facilities was 27.5 years. In terms of human resources at the facilities, the health centres had midwives, community health nurses (CHNs), and other types of staff, while CHPS facilities had no midwives but had CHNs and other staff. Most health workers at the facilities were women. All the study participants at the health centres were females.

**Table 1:** Background characteristics of health workers in participating health facilities, Northern Ghana, 2019 *Other Staff: were made up of enrolled nurses (lower calibre of general nurses), a nutrition officer, and a disease control officer HC: Health Centre; CHPS: Community Health Planning and Services; CHNs: Community Health Nurses (community health nurses have a 3-year diploma qualification)

Type of Health Facility	Mean Age Per Facility	Caliber of Staff		Sex		Total
				Female	Male	
HC1	33.67	Midwives	3	3	0	3
		CHNs	3	3	0	3
HC2	40.66	Midwives	3	3	0	3
		CHNs	1	0	1	1
		Other Staff*	2	0	2	2
CHPS1	25.00	Midwives	0	0	0	0
		CHNs	1	1	0	1
		Other Staff*	0	0	0	0
CHPS2	28.50	Midwives	0	0	0	2
		CHNs	1	1	0	1
		Other Staff*	1	1	0	0
CHPS3	27.50	Midwives	0	0	0	0
		CHNs	1	1	0	1
		Other Staff*	1	0	1	0
CHPS4	26.75	Midwives	0	0	0	0
		CHNs	2	1	1	2
		Other Staff*	2	0	2	2
CHPS5	29.50	Midwives	0	0	0	0
		CHNs	1	1	0	1
		Other Staff*	1	0	1	1
Clinic	28.00	Midwives	0	0	0	0
		CHNs	1	1	0	1
		Other Staff*	1	0	1	0

Table 2 shows the League Table for the three rounds of the mystery client surveys, and the results show consistent improvement in the services based on the actionable points in consecutive rounds. In Round 1, most facilities performed poorly (0 or 1) with CHPS 2, scoring the highest (that is, scoring ‘3’ in nine of the thirteen actionable points). HC1 and Clinic were the worst-performing health facilities in Round 1. In Round 2, more health facilities scored ‘3’; every facility obtained at least one ‘3’. Round 3 showed a further increase in the number of ‘3’ scores. CHPS5 was the worst-performing facility. The actionable points with the high scores across the three rounds were "warm reception", "FP room not labeled", "In-depth commodity explanation is required", "Offer clients a seat", and "Availability of commodity". On the other hand, the actionable points with low scores were "Identify wandering client", and "FP prevent pregnancy but not STIs except condoms".

**Table 2:** League table of health facility scoring for the three rounds of mystery client survey in Northern Ghana, 2019 HC: health centre; CHPS: community health planning and services; CHNs: community health nurses; FP: family planning; 24/7: 24 hours, 7 days a week; UPT: urine pregnancy test; STIs: sexually transmitted infections

Actionable points	First Round									Second Round									Third Round								
	HC1	HC2	CHPS1	CHPS2	CHPS3	CHPS4	CHPS5	Clinic	Total score	HC1	HC2	CHPS1	CHPS2	CHPS3	CHPS4	CHPS5	Clinic	Total score	HC1	HC2	CHPS1	CHPS2	CHPS3	CHPS4	CHPS5	Clinic	Total score
Warm reception	2	3	3	3	2	3	3	2	20	2	2	2	3	2	2	2	1	16	2	3	3	2	2	3	3	3	21
Confidentiality and Privacy	1	2	3	3	1	2	2	1	15	3	3	2	3	2	2	3	1	19	3	3	3	3	3	3	3	1	22
FP room not Labled	3	2	3	3	2	2	2	2	19	2	3	2	3	3	3	3	2	21	3	3	3	3	3	3	3	0	21
Service available 24/7	2	1	3	2	0	1	1	0	11	3	1	3	1	1	1	2	1	13	3	3	3	2	2	2	3	3	21
I dentify wandering clients	2	2	0	1	0	0	0	0	5	2	0	2	0	0	0	0	0	4	2	3	2	2	1	2	0	2	14
Any FP service provider can offer FP	1	0	1	2	0	1	1	1	7	3	3	3	2	1	1	2	2	17	3	3	3	1	2	3	0	2	17
UPT not being an obstacle	0	2	0	3	2	2	2	2	12	3	1	0	3	3	3	3	3	19	2	3	3	1	2	3	3	0	17
Rule out pregnancy	0	2	1	3	2	1	1	1	12	3	3	1	1	1	2	2	3	16	3	3	3	3	3	3	0	0	18
In-depth commodity explanation is required	1	3	2	3	1	1	2	1	15	3	3	3	3	3	2	3	3	23	2	3	3	2	1	2	3	3	19
FP prevent pregnancy but not STIs except condoms	0	2	0	2	0	0	0	0	5	0	0	0	0	0	1	1	2	4	3	3	0	2	1	1	1	2	13
Offer clients a seat	1	0	1	3	0	1	1	1	8	3	2	3	3	3	2	3	1	20	3	3	3	3	3	3	2	3	23
Availability of preferred FP commodity	3	2	3	3	3	3	3	0	20	3	3	3	3	0	2	0	3	17	3	3	3	2	2	3	3	3	22
Documentation	0	1	3	3	3	0	3	0	13	3	3	3	3	-	2	0	3	17	3	3	3	3	0	3	0	0	15

**Figure 1:**
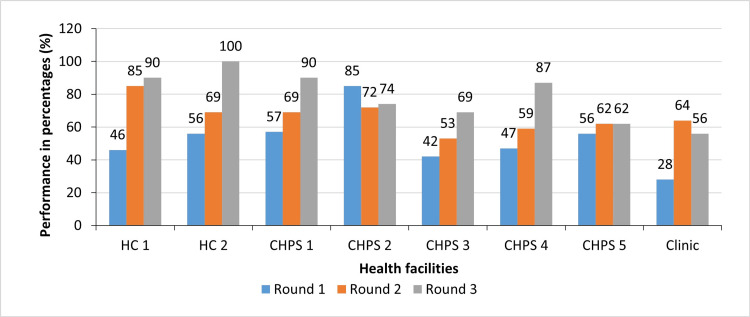
Performance of health facilities in percentages for the three rounds of mystery client surveys in Northern Ghana, 2019 HC: Health Centre; CHPS: Community Health Planning and Services

Figure 1 shows that five out of the eight health facilities improved in performance consistently for the three rounds. Particularly, health centres showed the greatest performance. 

**Figure 2:**
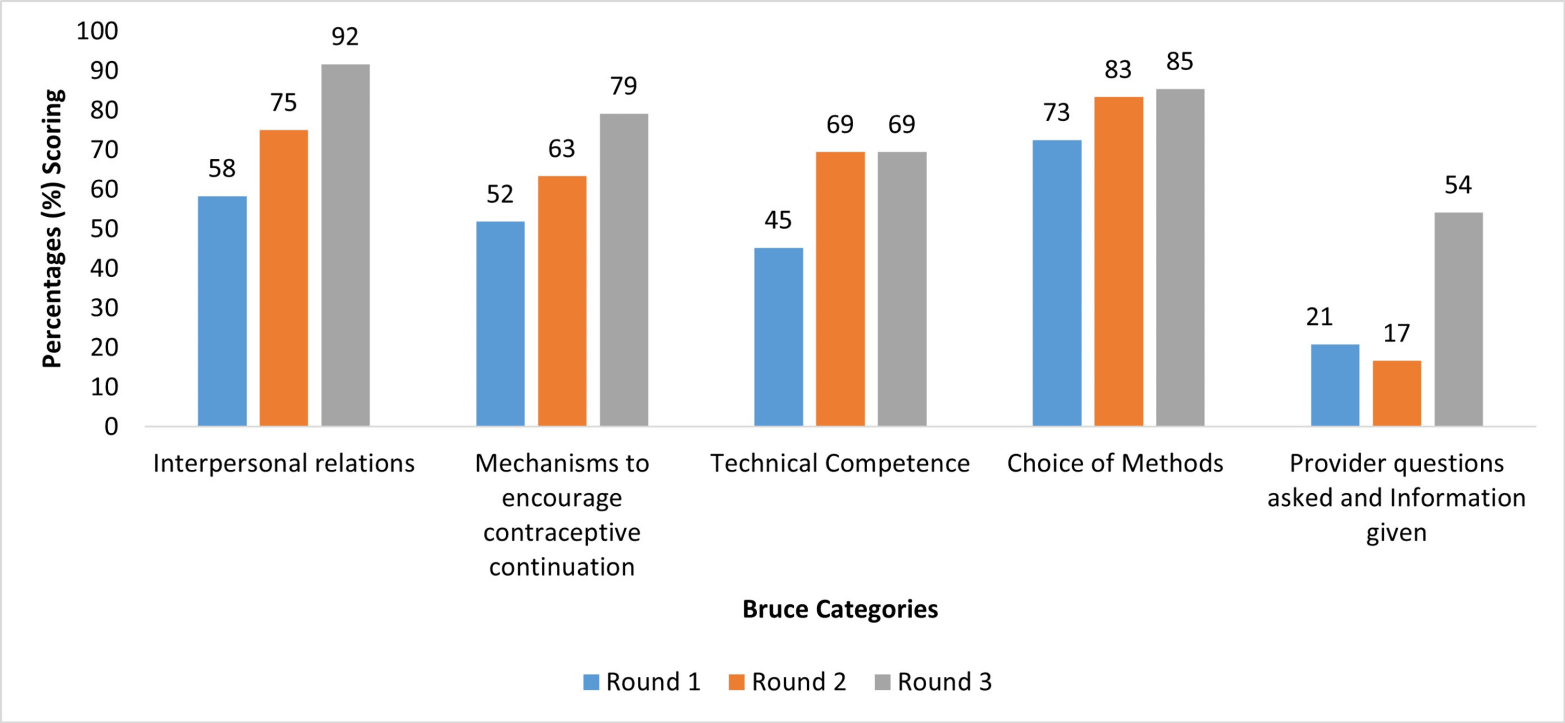
Percentage scoring by Bruce categorisation for mystery client survey in Northern Ghana, 2019

Figure 2 shows that performance improved in all of Bruce’s categories in consecutive rounds. However, the category of ‘Providers: Questions asked/ information given’ decreased in performance between the first and second rounds but markedly improved between the second and third rounds. Additionally, ‘Technical competence’ did not change in performance between Rounds 2 and 3.

Table 3 shows the total scoring obtained by facilities for the three rounds. In general, the total scoring increased for consecutive rounds. The increase in mean scores was not significant between consecutive rounds. Thus, between Round 1 (20 (±6.9)), p=0.0554, and Round 2 (25.75 (4.1)), p=0.0474, and Round 3 (30.38 (±6.2)), p=0.0084. However, the increase in mean scores was significant between Rounds 1 and 3, p=0.0084. The repeated-measures ANOVA test was also significant for the increase in mean scoring for the three rounds (p=0.0032).

**Table 3:** Health facility performance of family planning services received by mystery clients in Northern Ghana, 2019 HC: health center; CHPS: community health planning and services; ANOVA: analysis of variance

Health Facilities	Round 1		Round 2		Round 3	
	Score	Percentage (%)	Score	Percentage (%)	Score	Percentage (%)
HC1	16	41	33	85	35	90
HC2	22	56	27	69	39	100
CHPS1	23	59	27	69	35	90
CHPS2	34	87	28	72	29	74
CHPS3	16	41	19	49	25	64
CHPS4	17	44	23	59	34	87
CHPS5	21	54	24	62	24	62
Clinic	11	28	25	64	22	56
Total	160		206		243	
Mean Score (SD)	20 (±6.9)	51	25.75 (±4.1)	66	30.38 (±6.2)	78
Significance level between Rounds Using Paired t-test between rounds (p-value)	Round 1 and Round 2		Round 2 and Round 3		Round 1 and Round 3	
	0.0554		0.0474		0.0084	
ANOVA test: Three Rounds	0.0032					
Assumptions for ANOVA and t-test	Test for normality using the Shapiro-Wilk test: p-value=0.88091. Test for variance using Mauchly’s test: p-value=1. Test for independence using a scatter diagram, which showed that the points were scattered, indicating the independence of the observations in the dataset.					

**Figure 3:**
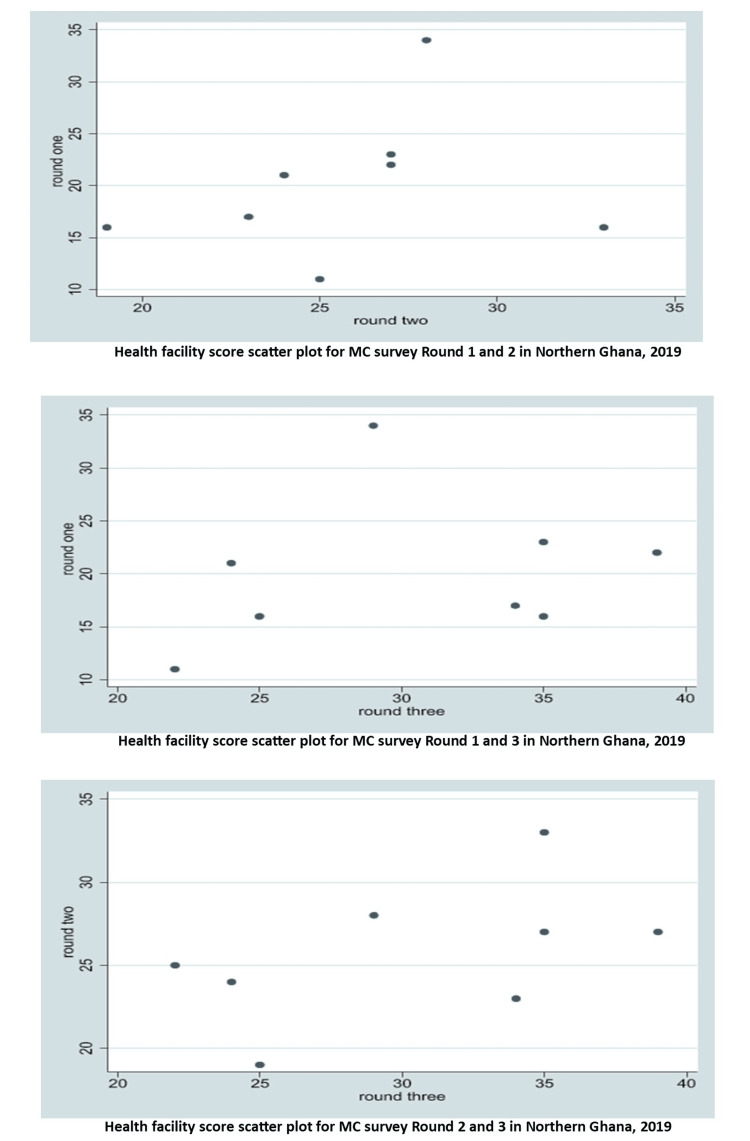
Health facility score scatter plots for mystery client survey between rounds in Northern Ghana, 2019

Figure 3 displays the scatter plots for MC surveys between rounds, illustrating the independence of the dataset used for the analysis of Figures 1, 2 and Table 3. 

Table 4 provides information on findings from the MC survey feedback sessions in Northern Ghana. They include possible identification of MCs by health workers, reactions of health workers to their performance, and the district health authorities' decision to adopt the MC approach into their monitoring strategies. 

**Table 4:** Findings from content analysis of MC survey feedback sessions in Northern Ghana, 2019 FP: Family Planning, CHPS: Community Health Planning and Services, HC: Health Centre, MC: Mystery Client, STIs: Sexually Transmitted Infections

Themes	Findings	Quotes (Codes)
Health workers' suspicions of the presence of MC result in adherence to actionable points.	Several health workers indicated that they had identified that some of their clients were different from those they usually saw. They therefore suspected that those clients were MCs. This was particularly the case in Round 1. This finding became apparent during the discussions at the feedback session for the first round. Health workers mentioned that they will be more vigilant and treat every client as a mystery client. Given this, they eventually mentioned that they are going to take the 13 actionable points seriously.	“A community health nurse commented that she suspected one woman who came to her facility and reported that she had come to visit the family, but she wanted to use the opportunity to do FP outside her community. Because of this, she was going to treat every FP client as a potential MC.” (Feedback session 1)
Health workers' reactions to performance	Due to the health workers’ experience from the first feedback, health workers became more conscious of adhering to the 13 actionable points. The healthcare workers were, therefore, expecting higher performance in the second feedback session, but some of them were disappointed with their performance.	"A community health nurse even argued that she did not understand why her facility was scored ‘1’ for offering a client a seat, because the facility had chairs for clients in the consulting room. She was then given the response by the investigative team member that the MC had reported that the health care worker who attended to her was engaged in another activity while welcoming the MC and did not offer her the seat until the health care worker was done with that activity. The MC further commented that the health worker took about 2 minutes before offering her the seat.” (Feedback session 2)
Reasons for low performance in some actionable points	Health workers gave reasons why two actionable points (“Identifying a wandering client” and “FP prevents pregnancy but not STIs except condoms”) performed poorly in the Round 1 and Round 2 surveys.	“When health workers were asked why they still had low scores for not identifying wandering clients, since this was the second mystery client survey, a health worker explained that ‘Clients’ relatives mostly sit within the facility premises, but outside the building, so it was difficult to distinguish between a wandering client and a client’s relative.” (Feedback session 2- Health worker, HC) “Health workers also complained that the workload is too much, so they didn’t have the ‘luxury’ of time to explain things to clients in detail.” (Feedback session 2)
Proposed solutions to low-performing actionable points	Both health authorities and health workers agreed that health workers can approach suspected clients discreetly and inquire whether they are coming for services.	“A member of the District health authorities instructed the health workers to be more inquisitive about the people who always hang around the facility and approach them to see if they needed any service.” (Feedback session 3)
Health authorities' decision to adopt the Mystery Client approach among routine monitoring strategies	The district health authorities observed that the MC survey was contributing to the improvement of service quality and decided to incorporate it into their monitoring strategy after the research period had ended. Therefore, they announced it to the health workers.	“The District health authorities further informed the health workers that they will be visiting their facilities unannounced to ensure that the health workers were adhering to the 13 actionable points and would continue to use the services of mystery clients even after the research was completed.” (Feedback session 3)

## Discussion

The importance of providing quality family planning services by health workers is well known [2, 23-24]. Findings from this study show an improvement in the quality of care given by health facilities over the three MC surveys based on the 13 actionable points. Additionally, in terms of actionable points, all the groups of the Bruce categorisation [21] showed an improvement except ‘Provider: Questions asked and information given’. Furthermore, the results indicated that the mean scores increased across the three consecutive rounds of MC surveys with feedback to health workers. This suggests an improvement in the quality of care, as observed particularly between Rounds 1 and 3.

The finding that MC methods improve the quality of care aligns with the literature [16]. This is particularly found in repeated MC visits. For example, Collins et al [25], using repeated mystery clients as an interventional study in Australia, identified that with 521 repeated visits to pharmacy shops by mystery clients, there was an overall improvement in outcomes by 54% (p=0.001) in the management of minor ailments. Their finding align with our results in that repeated mystery client visits to participating facilities result in improvement in study outcomes.

The improvement in the quality of care resulting from the consecutive rounds of the MC survey could be attributed to the feedback given. For instance, Collins et al observed that repeated MC visits with feedback improved study outcomes [25]. This finding supports our results. The use of feedback to improve the quality of care was also observed by Krägeloh et al [26], who used feedback from patients to improve the quality of care. If feedback from patients is beneficial, then the information gathered from MC surveys given as feedback is even more advantageous because the information was gathered in a real-life health care setting. This is because feedback from MC surveys is not based on the responses of the health workers, who may hide important or sensitive information. This finding is supported by the works of Ganle et al in Ghana [17] and Footman et al's systematic reviews in low- and middle-income countries [27]. Additionally, we gathered information about the service delivery processes [28].

Also, Argaw et al [29] stated that when there is support from authorities, the technical competency of health workers is improved through coaching. During the feedback sessions, the district health authorities provided support by coaching. Furthermore, when district authorities visited the facilities, the issues raised during the meetings were reiterated.

In terms of the Bruce categorisation of the actionable points, one category that showed an improvement in the successive rounds was that clients received their ‘choice of contraceptives’. The improvement in the "choice of contraceptives" category was attributed to MCs being encouraged to choose combined oral contraceptives (COCs), as they are non-invasive and require minimal provider skills. Fortunately, COCs were available at the time of the surveys, making them a viable and acceptable option. Despite these successes, all the health facilities, except HC2, performed poorly in identifying wandering clients. This was attributed to the inability of health workers to distinguish between patients' relatives and wandering clients, as reported by health workers during feedback sessions. This was particularly disconcerting because it had been established by the larger study (outside the scope of this article) that low contraceptive uptake could be attributed to a prevailing stigma associated with modern contraception [30]. From the feedback sessions, health authorities directed that health workers should be curious enough to approach any suspected client to inquire whether they need services. Therefore, a future intervention should emphasise identifying such hesitant and unsure clients due to stigma.

The study also showed the number of staff in each category who participated in the training from the health facilities. Community health nurses averaged two in the CHPS facilities, while midwives in each health centre also averaged three. According to the Staffing Norm for the Health Sector, these numbers were adequate for the facilities. Since there was an improvement in the quality of services rendered, it implies that the number of health workers may not have negatively impacted the performance of facilities. 

Finally, the findings show that the consecutive use of MC surveys with feedback to health workers improves the quality of family planning services based on the 13 actionable points. Researchers and decision-makers should consider using MC surveys with feedback as part of quality improvement efforts and to collect information on the quality of service delivery in real-life settings. 

Study strengths and limitations

The study had a number of strengths as follows: Three rounds of MC surveys with feedback were used to assess and contribute to improving the quality of service delivery. The strategy used assessed the quality of service in a real-life healthcare environment. The MC surveys with feedback can be an effective and adaptable way to improve healthcare systems. The study also used district health officials to conduct the evaluations, and believed that this improved the quality of evidence from the study. 

One cannot, however, completely attribute an improvement in outcome to the intervention put in place because the study could not have a comparator to draw an inferential analysis. However, the grading of health facilities improved; therefore, it showed that the mystery client survey with feedback to health workers as an intervention was effective in improving the quality of care. 

We also acknowledge that different people may observe things differently. Therefore, grading may have lacked uniformity. However, observer bias was reduced by the use of the standardized checklist developed from the 13 actionable points. 

Furthermore, the inclusion of HC2 and CHPS5, even though they did not benefit from the health workers' training, could influence the comparability of performance.

A further limitation of this study was the limited time span between the Rounds 2 and 3 surveys. Thus, the findings of the improvement in performance between the MC surveys could have been biased. 

Additionally, the use of different types of MCs may have given different perspectives and biased the results, particularly between Rounds 1 and the remaining rounds. However, the computing of the scores was done by the district authorities and the investigative team, ensuring similar scoring.

Moreover, different calibers of health workers may have influenced the ability to adhere to the actionable points, thus risking the introduction of possible bias into the study.

Finally, the limited number of participating facilities in the study may affect the generalizability of the study.

## Conclusions

Family planning services improved in participating health facilities over the three consecutive rounds of MC surveys, linked to feedback to health workers. Additionally, there was an improvement in actionable points, particularly the “choice of contraceptives”. It is therefore recommended that mystery client surveys coupled with feedback to health workers could be used as a tool to improve the quality of service in family planning facilities.

Future research should take cognizance of the different caliber of service providers. Also, future studies may be challenged by identifying MC candidates with similar demographic characteristics, such as educational level and socioeconomic status. This is needed to reduce bias. Additionally, future studies may also be hampered by the availability of financial and other resources that may be required to train mystery clients and conduct the survey.

## Appendices

**Table 5:** Health facility assessment sheet FP: Family Planning, STIs: Sexually Transmitted Diseases, UPT:  Urine Pregnancy Test

Health facility performance assessment sheet		
S/N	Actionable points	Score
1	Health worker should know that we are service providers and not service commanders (warm reception)	
2	All family planning clients should be given the highest level of CONFIDENTIALITY and PRIVACY and ensure that their identity is protected	
3	There should NOT be an openly identified place or room for family planning services so as to prevent stigma	
4	Health worker should let clients know that they can visit the health facility any time they wish to offer family planning services	
5	Health workers should always look out for clients who may find it difficult to enter the health facility and may be wandering around	
6	Inform client that any family planning service provider can offer family planning services not only the service provider met at the first time	
7	Health workers should find an alternative means to determine the eligibility of the client if she cannot afford UPT in other to prevent miss-opportunities	
8	Health works should examine clients very well in other to be sure that there is no conception before offering family planning services	
9	In-depth explanation on all contraceptives should be given to clients in other to help the client choose a desired method and avoid change of decision	
10	Clients should be made aware that contraceptives are only used to prevent pregnancy but not STIs except condoms	
11	Be reminded that clients should be a offered a seat to feel relaxed before talking to them	
12	Availability of preferred FP commodity	
13	Documentation	
Total Health facility score		
% score for Health facility		
Scale		
0	Action not performed/observed	
1	Poorly performed	
2	fairly performed	
3	Perfectly performed	
